# The SLC Family Are Candidate Diagnostic and Prognostic Biomarkers in Clear Cell Renal Cell Carcinoma

**DOI:** 10.1155/2020/1932948

**Published:** 2020-05-01

**Authors:** Weiting Kang, Meng Zhang, Qiang Wang, Da Gu, Zhilong Huang, Hanbo Wang, Yuzhu Xiang, Qinghua Xia, Zilian Cui, Xunbo Jin

**Affiliations:** ^1^Department of Urology, Shandong Province Hospital Affiliated to Shandong University, 324 Jingwuweiqi Road, Jinan, Shandong 250000, China; ^2^Department of General Surgery, The Second People's Hospital of Dongying, 28 Changchun Road, Dongying, Shandong 257000, China; ^3^Department of Human Resources, Shandong Province Hospital Affiliated to Shandong University, 324 Jingwuweiqi Road, Jinan, Shandong 250000, China; ^4^Department of Plastic Surgery, Jinan Central Hospital Affiliated to Shandong University, 105 Jiefang Road, Jinan, Shandong 250000, China; ^5^Department of Urology, Lanling People's Hospital, 4 Jiankang Street, Lanling, Shandong 277700, China

## Abstract

Clear cell renal cell carcinoma (ccRCC) is the most common lethal subtype of renal cancer, and changes in tumor metabolism play a key role in its development. Solute carriers (SLCs) are important in the transport of small molecules in humans, and defects in SLC transporters can lead to serious diseases. The expression patterns and prognostic values of SLC family transporters in the development of ccRCC are still unclear. The current study analyzed the expression levels of SLC family members and their correlation with prognosis in ccRCC patients with data from Oncomine, Gene Expression Profiling Interactive Analysis (GEPIA), The Cancer Genome Atlas (TCGA), cBioPortal, the Human Protein Atlas (HPA), the International Cancer Genome Consortium (ICGC), and the Gene Expression Omnibus (GEO). We found that the mRNA expression levels of SLC22A6, SLC22A7, SLC22A13, SLC25A4, SLC34A1, and SLC44A4 were significantly lower in ccRCC tissues than in normal tissues and the protein expression levels of SLC22A6, SLC22A7, SLC22A13, and SLC34A1 were also significantly lower. Except for SLC22A7, the expression levels of SLC22A6, SLC22A13, SLC25A4, SLC34A1, and SLC44A4 were correlated with the clinical stage of ccRCC patients. The lower the expression levels of SLC22A6, SLC22A13, SLC25A4, SLC34A1, and SLC44A4 were, the later the clinical stage of ccRCC patients was. Further experiments revealed that the expression levels of SLC22A6, SLC22A7, SLC22A13, SLC25A4, SLC34A1, and SLC44A4 were significantly associated with overall survival (OS) and disease-free survival (DFS) in ccRCC patients. High SLC22A6, SLC22A7, SLC22A13, SLC25A4, SLC34A1, and SLC44A4 expression predicted improved OS and DFS. Finally, GSE53757 and ICGC were used to revalidate the differential expression and clinical prognostic value. This study suggests that SLC22A6, SLC22A7, SLC22A13, SLC25A4, SLC34A1, and SLC44A4 may be potential targets for the clinical diagnosis, prognosis, and treatment of ccRCC patients.

## 1. Introduction

Kidney cancer is one of the most common malignant tumors in the urinary system, and more than half of patients are found by chance and have no clinical symptoms. In 2019, the number of new cases of kidney cancer in the United States was 73,820, ranking fifth in the number of new cases in men and eighth in the number of new cases in women [1]. Renal cell carcinoma (RCC) is the most common form of kidney cancer and accounts for 90% of all tumors, with ccRCC being the most common histology (75%) [2, 3]. ccRCC is derived from the proximal tubular epithelium of the kidney and exhibits unique histological characteristics. Macroscopically, ccRCC are golden yellow in section and often have bleeding, necrosis, and cystic areas. Histologically, ccRCC usually consists of tumor cells with clear cytoplasm, surrounded by nests or tubules with a rich network of blood vessels. The clear appearance of the cytoplasm is due to the accumulation of glycogen and lipids [4]. Different renal cancer cell subtypes have different genetic changes and different prognoses. The cure rate is high for patients with early, localized disease, with 5-year survival at more than 90% [5]. In contrast, 5-year survival drops to 12% for patients with distant metastatic disease.

Epigenetic changes in ccRCC may be related to its prognosis and treatment. Genetic markers of ccRCC include biallelic inactivation of Von Hippel-Lindau (VHL) tumor suppressor genes; negative regulators of hypoxia-inducible factor (HIF) protein; copy number changes of chromosome 3p, 5q, and 14q genes; and chromatin-modifying enzyme high mutation frequencies, such as protein polybromo-1 (PBRM1), SET domain containing 2 (SETD2), and BRCA1-associated protein-1 (BAP1) [6]. Currently, multitarget tyrosine kinase inhibitors (TKIs) and mechanistic target of rapamycin kinase (mTOR) inhibitors have become major breakthroughs in the treatment of ccRCC. In addition, immune checkpoint inhibitors have also become effective treatment options against advanced ccRCC. However, ccRCC has been called “metabolic disease,” and its multiple bioenergy pathways are changed [7, 8]. Extensive metabolic reprogramming in glucose, lipid, and amino acid metabolism has largely promoted the clear cell phenotype [9], but the genetic mechanism of these changes is not fully understood.

In recent years, the role of membrane transporters in cancer has received increasing attention. Two superfamily transporters have been discovered, namely, the ATP-binding cassette (ABC) family and SLC family. SLC transporters help ingest certain essential molecules [10], such as amino acids and glucose, and regulate metal absorption, mediating the normal functions of important enzymes [11]. Previous studies have found several essential nutrient transporters, such as the glucose transporter SLC2A1 [12] and the amino acid transporters SLC1A5, SLC7A5, SLC6A14, SLC7A11, and SLC38A2 [13–17], which are upregulated in cancer as tumor promoters. SLC39A1 regulates the malignant potential of prostate cancer cells by inhibiting the nuclear factor kappa B (NF-*κ*B) signaling pathway, and SLC39A1 may play a role as a tumor suppressor gene in prostate cancer [18]. A good understanding of the differential expression of SLC transporters in various cancer cells can provide a theoretical basis for the development of new strategies for the treatment of cancer.

Although some scholars have studied the differential expression of SLC mRNA in ccRCC, it was found that SLC10A2 was significantly downregulated in TKI-treated samples and SLC10A2 was upregulated in ccRCC compared with adjacent kidney tissues in paired The Cancer Genome Atlas (TCGA) samples. High SLC10A2 expression was associated with good prognosis of ccRCC [19]. The results were contradictory. In this study, a bioinformatics analysis was performed to explore the roles of multiple SLCs in ccRCC. We analyzed the expression and mutation of different SLCs in ccRCC patients to determine their expression patterns, potential functions, and prognostic values in ccRCC.

## 2. Materials and Methods

### 2.1. Screening Cancer-Associated Genes in ccRCC and the Expression Level of the SLC Family Genes in Pancancer Using Oncomine Datasets

The Oncomine datasets (http://www.oncomine.org) integrate RNA and DNA sequencing data from sources such as the Gene Expression Omnibus (GEO), TCGA, and published literature. We used the Jones Renal dataset to screen overexpressed and underexpressed genes in the associated concept tab of Oncomine. Compared with normal tissues, we screened top 10% overexpressed and 10% underexpressed genes in Jones Renal dataset of ccRCC. These screened genes were used in the next step of data analysis.

We use Oncomine to analyze the expression levels of the SLC family genes in pancancer (cancer vs. normal). The *P* value was defined as 0.01, fold change (FC) was defined as 2, gene rank was defined as all, and data type was also defined as all.

### 2.2. The Differential Expression and Prognostic Value of the SLC Family Genes Using Gene Expression Profiling Interactive Analysis (GEPIA) Dataset

GEPIA (http://gepia.cancer-pku.cn/) is a newly developed interactive web server for analyzing the RNA sequencing expression data of 9,736 tumor samples and 8,587 normal samples from TCGA and Genotype-Tissue Expression (GTEx) projects using a standard processing pipeline [20]. We used GEPIA to analyze the differential expression of the SLC family in ccRCC and its relationship with clinical stage. TCGA normal tissue and GTEx normal tissue were matched. The *P* value and fold change were defined as 0.01 and 2, respectively. In addition, the GEPIA was used to analyze the correlation between the SLC family and the prognosis of ccRCC. The group cutoff for the survival analysis was 50%, and the *P* value and fold change were defined as 0.01 and 2, respectively.

### 2.3. Analysis of Genetic Alteration in the SLC Family Genes Using cBioPortal

TCGA (http://www.cancer.gov/tcga) collected, characterized, and analyzed cancer samples from over 11,000 patients over a 12-year period. TCGA data, resources, and materials were originally published by the National Cancer Institute. We used the cBioPortal tool to analyze 538 cases of kidney renal clear cell carcinoma (TCGA, Firehose Legacy). The cBioPortal for cancer genomics (http://www.cbioportal.org/) provides the visualization, analysis, and download of large-scale cancer genomic datasets [21, 22]. The genomic profiles included mutations, putative copy number alterations from the Genomic Identification of Significant Targets in Cancer (GISTIC), mRNA expression *Z*-scores (RNASeq V2 RSEM), and protein expression *Z*-scores (RPPA). In addition, the correlation between the expression levels of the SLC family genes was analyzed using a plot module of cBioPortal. Data type was selected mRNA and the mRNA profile was selected mRNA expression *Z*-scores (RNASeq V2 RSEM). Then, we used the bioconductor “pheadmap” package for drawing.

### 2.4. Detection of the SLC Family Protein Expression in Tissues by the Human Protein Atlas (HPA)

The HPA was a Swedish-based programme initiated in 2003 with the aim of mapping all the human proteins in cells, tissues, and organs using the integration of various -omics technologies, including antibody-based imaging, mass spectrometry-based proteomics, transcriptomics, and systems biology. Immunohistochemistry (IHC) images of ccRCC were kindly provided by the Protein Atlas Project, which is publicly available (http://www.proteinatlas.org). In the Human Protein Atlas, all images of tissues stained by immunohistochemistry were manually annotated by a specialist followed by verification by a second specialist. Staining intensity was divided into four levels: negative, weak, medium, and strong. According to the fraction of stained cells, staining quantity was also divided into four levels: none, <25%, 25-75%, and >75%. Protein expression levels were based on staining intensity and staining quantity. The classification criteria for protein expression levels were as follows: negative, not detected; weak and <25%, not detected; weak combined with either 25-75% or 75%, low; moderate and <25%, low; moderate combined with either 25-75% or 75%, medium; strong and <25%, medium; and strong combined with either 25-75% or 75%, high.

All slides with SLC22A6, SLC22A7, SLC22A13, SLC25A4, SLC34A1, and SLC44A4-staining were downloaded from the HPA. The average number of normal tissues was 5, and the average number of cancer tissue was 36.8. The protein expression levels in cancer and normal tissues were compared in terms of staining, intensity, and quantity. The data was subjected to a Mann–Whitney *U* test using SPSS to calculate the *P* value. When performing statistical calculations, the weight of each patient was 1. If a patient had multiple samples, 1 was equally divided into each sample. The *P* value was defined as 0.01.

### 2.5. The Protein-Protein Interaction (PPI) Network Construction and Analysis of Modules

The STRING database (http://http://string-db.org/) provides a significant association of protein-protein interactions [23]. Cytoscape is used for the visual exploration of interaction networks [24]. In this study, PPI networks were analyzed by the STRING database and subsequently visualized by using Cytoscape. The minimum required interaction scores were defined as high confidence (0.700). The max number of interactors to show was set as 100. Cytoscape was then used to visualize the PPI network. The Cytoscape plugin Molecular Complex Detection (MCODE) was used to screen out modules of PPI networks, and the degree cutoff = 2, node score cutoff = 0.2, *k* − score = 2, and max depth = 100 [25].

### 2.6. Metascape for Functional Enrichment and Kyoto Encyclopedia of Genes and Genomes (KEGG) Analysis

The aim of Metascape (http://metascape.org/gp/index.html) is to develop a set of reliable, productive, and intuitive tools that help a biomedical research community to analyze gene/protein lists and make better data-driven decisions. Metascape combines feature enrichment, cross analysis, gene annotation, and other search functions, including more than 40 independent knowledge bases [26]. We used Metascape to perform functional enrichment and KEGG analysis of SLC family member protein and its interacting proteins. Only terms with *P* < 0.01, a minimum count of 3, and an enrichment factor > 1.5 were considered significant.

### 2.7. Verify the Differential Expression of the SLC Family Genes Using GSE53757 Dataset

The data of ccRCC were derived from the GEO. We used the Bioconductor “GEOquery” package to download the GSE53757 dataset from the GEO. Subsequently, differential expression analysis was performed using the Bioconductor “limma” package. The *P* value and fold change were defined as 0.01 and 2, respectively. Then, the bioconductor “pheatmap” package was used to draw the heat map and the bioconductor “ggplot” package was used to draw the boxplot.

### 2.8. Validate the Prognostic Value of the SLC Family Genes Again Using the International Cancer Genome Consortium Dataset

The package “UCSCXenaTools” of R software was used to download the expression data and clinical data of ccRCC from the ICGC. The downloaded dataset included a total of 594 specimens, of which 72 were normal tissues and 30 patients had a survival time of less than 30 days. A total of 492 ccRCC patients were included for survival analysis with the R package “survival.” The *P* value was defined as 0.01.

## 3. Results

### 3.1. Screening the SLC Family in ccRCC

**Figure 1:**
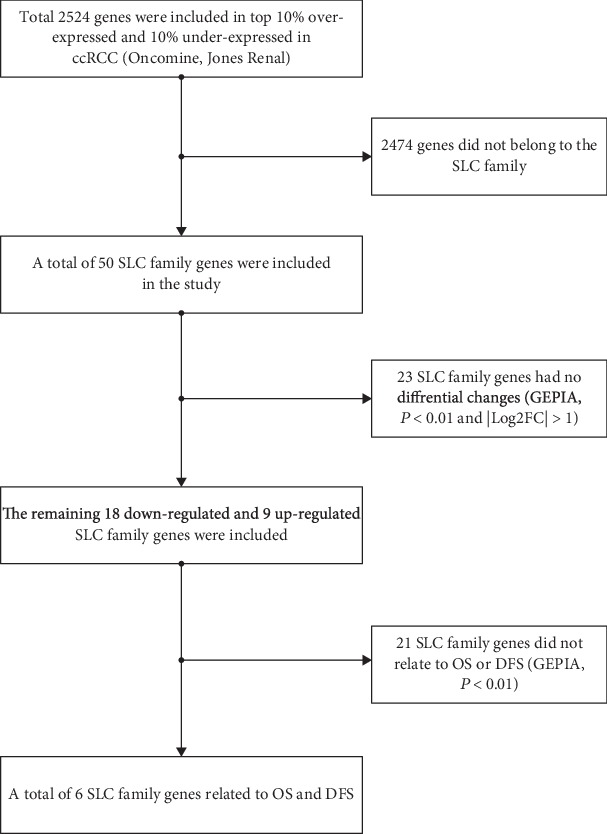
Screening process of the SLC family. OS: overall survival; DFS: disease-free survival.

First, Oncomine was used to select ccRCC in the Jones Renal database, which includes 23 pairs of cancer and normal tissues. The top 10% overexpressed genes and 10% underexpressed genes, totalling 2,524 genes, were selected, of which 50 were SLC family genes. Second, the SLC family genes with the differential expression in ccRCC were screened again using GEPIA. The *P* value and fold change were defined as 0.01 and 2, respectively. Third, GEPIA was used to screen SLC family genes associated with OS and DFS in ccRCC. The group cutoff for the survival analysis was 50%, and the *P* value and fold change were defined as 0.01 and 2, respectively. The specific process is shown in Figure 1. Finally, six SLC family genes were screened: SLC22A6, SLC22A7, SLC22A13, SLC25A4, SLC34A1, and SLC44A4.

### 3.2. The Expression Levels of SLC Family Genes in Pancancer

**Figure 2:**
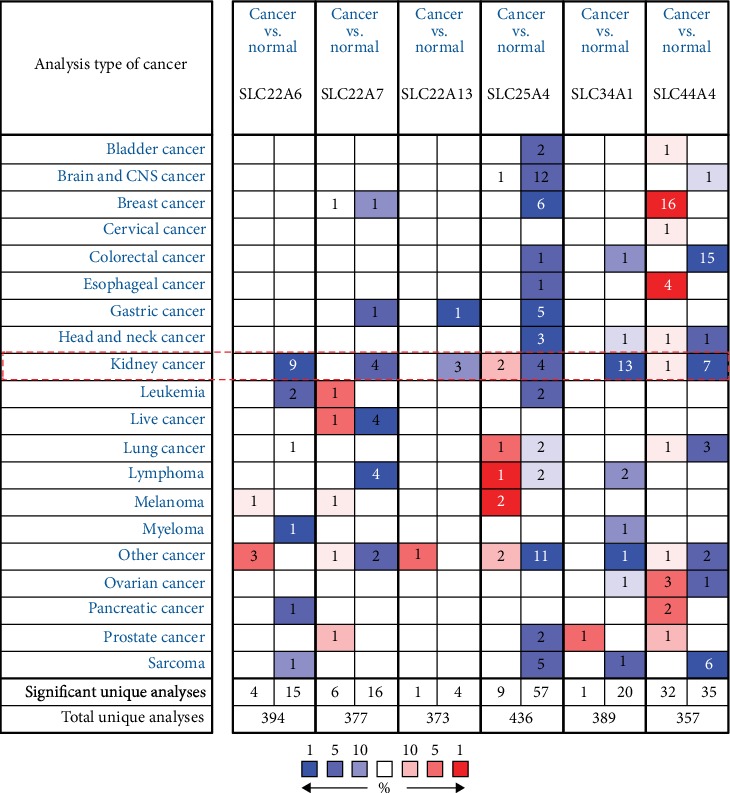
The expression levels of SLC family genes in pancancer (cancer vs. normal). The degree of cell color was determined by the best gene rank percentile. Red represented overexpression and blue represented underexpression. The number in the cell indicates the number of datasets that reached the threshold in the corresponding datasets of cancers.

We used the Oncomine database to analyze the expression levels of SLC22A6, SLC22A7, SLC22A13, SLC25A4, SLC34A1, and SLC44A4 in various cancers and their normal tissues (Figure 2). Among the various cancers examined, SLC22A6, SLC22A7, SLC22A13, SLC25A4, and SLC34A1 were significantly downregulated, while SLC44A4 showed different expression patterns in different cancers (upregulated or downregulated). In kidney cancer, SLC22A6, SLC22A7, SLC22A13, SLC25A4, SLC34A1, and SLC44A4 were significantly downregulated.

### 3.3. The Difference Analysis of SLC Family Genes and Its Relationship with Clinical Stage

**Figure 3:**
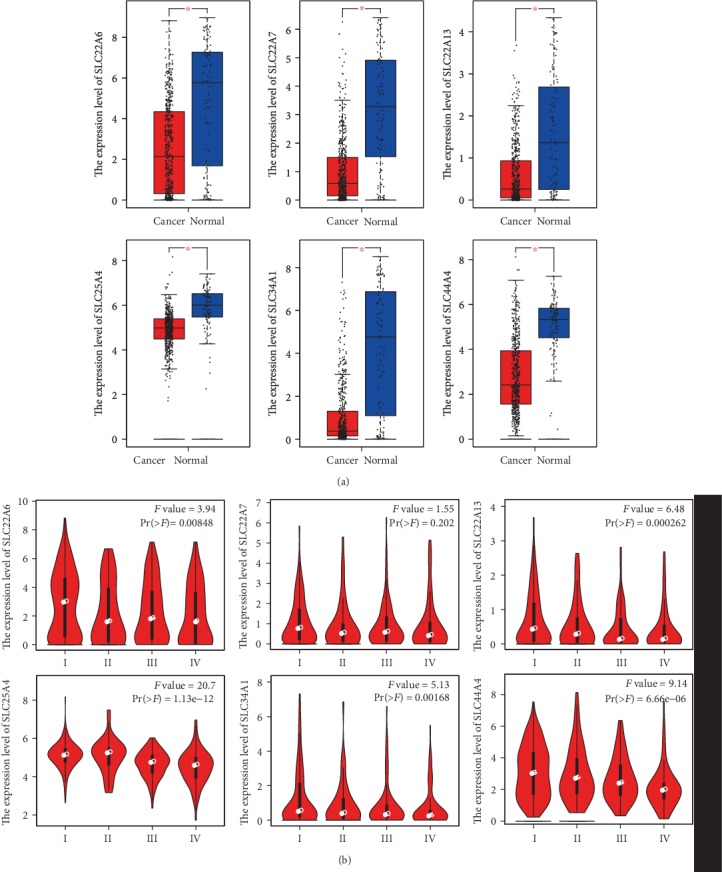
The expression level of SLC family genes and its correlation with tumor stage in ccRCC (GEPIA). (a) The boxplot of the SLC family gene expression level. ^∗^*P* < 0.01 and |Log2FC| > 1. (b) The correlation between the expression of SLC family genes and tumor stage (I, II, III, and IV) in ccRCC. Pr(>F) < 0.01 meant the difference was statistically significant.

The expression levels of SLC22A6, SLC22A7, SLC22A13, SLC25A4, SLC34A1, and SLC44A4 in ccRCC were compared using the GEPIA database. A total of 523 ccRCC cancer tissues and 100 normal tissues were included. As shown in Figures 3(a), the expression levels of SLC22A6, SLC22A7, SLC22A13, SLC25A4, SLC34A1, and SLC44A4 mRNAs in ccRCC were significantly decreased, and the difference was statistically significant (*P* < 0.01 and ∣Log_2_FC∣ > 1).

Further studies using the GEPIA database revealed that the expression levels of SLC22A6, SLC22A7, SLC22A13, SLC25A4, SLC34A1, and SLC44A4 in ccRCC correlated with the clinical stage of the cancer, as shown in Figure 3(b). The expression levels of SLC22A6, SLC22A13, SLC25A4, SLC34A1, and SLC44A4 were significantly correlated with the clinical stage of the cancer. The lower the expression levels of SLC22A6, SLC22A13, SLC25A4, SLC34A1, and SLC44A4 were, the later the clinical stage of the ccRCC patients was. There was no correlation between the expression level of SLC22A7 and the clinical stage of the cancer.

### 3.4. The Differential Expression of SLC Family Proteins Was Detected by Immunohistochemistry

**Figure 4:**
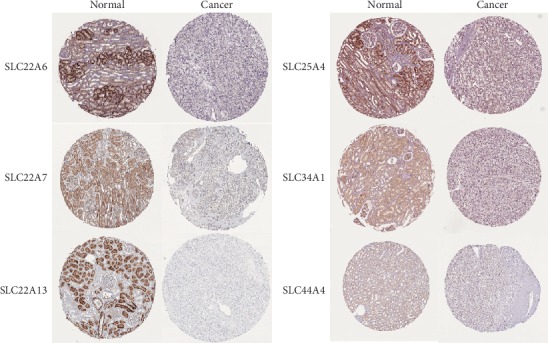
Representative tissue microarray (TMA) slides with SLC22A6, SLC22A7, SLC22A13, SLC25A4, SLC34A1, and SLC44A4 staining. All slides were retrieved from the Human Protein Atlas (HPA, http://www.proteinatlas.org). Magnification, ×100.

The expression levels of SLC22A6, SLC22A7, SLC22A13, SLC25A4, SLC34A1, and SLC44A4 proteins in ccRCC were studied using the HPA. We downloaded all slides stained with SLC22A6, SLC22A7, SLC22A13, SLC25A4, SLC34A1, and SLC4A4 from the HPA. A total of 30 normal tissues were included, and the average number of normal tissues per gene was 5. A total of 221 cancer tissues were included, and the average number of cancer tissues per gene was 36.8. The protein expression levels in cancer and normal tissues were compared in terms of staining, intensity, and quantity. Compared with normal tissues (as shown in Figure 4 and Table 1), the expression levels of SLC22A6, SLC22A13, and SLC34A1 proteins in ccRCC tissues were significantly decreased in terms of staining, intensity, and quantity. Compared with normal tissues, the expression levels of SLC22A7 proteins in ccRCC tissues were significantly decreased in terms of staining and quantity. In cancer tissues, SLC25A4 and SLC44A4 proteins did not change significantly in terms of staining, intensity, and quantity.

### 3.5. Association of SLC Family Genes with the Prognosis of Patients with ccRCC

**Figure 5:**
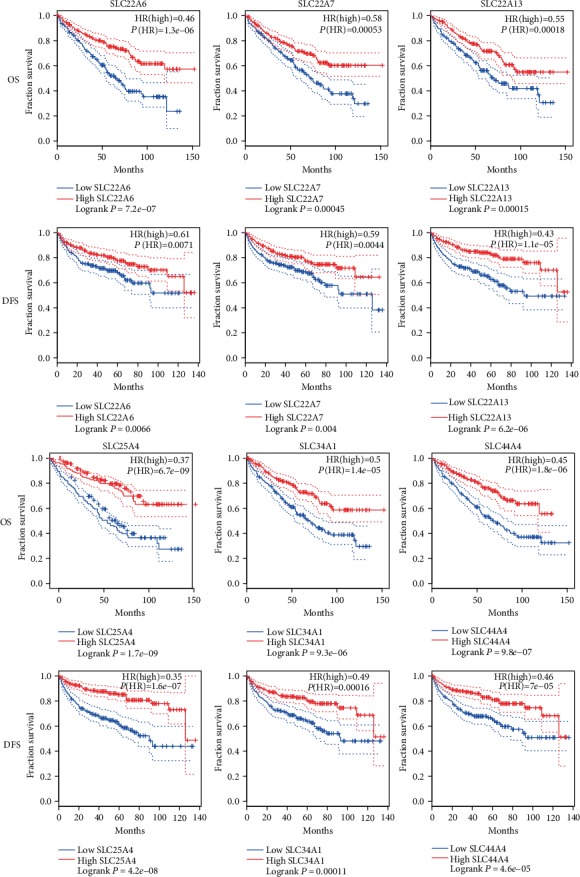
Kaplan-Meier survival curves for OS and DFS according to the expression level of SLC family genes in ccRCC (GEPIA). OS: overall survival; DFS: disease-free survival; HR: hazard ratio. Logrank *P* < 0.01 meant the difference was statistically significant.

The impact of SLC family genes on the survival of ccRCC patients was analyzed with the GEPIA database. As shown in Figure 5, the expression levels of SLC22A6, SLC22A7, SLC22A13, SLC25A4, SLC34A1, and SLC44A4 in ccRCC were significantly correlated with the OS and DFS of patients, and the difference was statistically significant (*P* < 0.01). High SLC22A6, SLC22A7, SLC22A13, SLC25A4, SLC34A1, and SLC44A4 expression predicted improved OS and DFS.

### 3.6. The Genetic Alterations of SLC Family Genes in ccRCC

**Figure 6:**
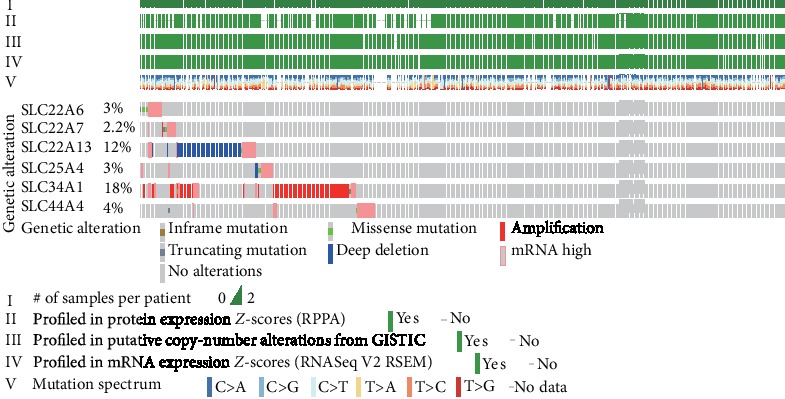
The genetic alteration analysis and the correlation of the SLC family genes in ccRCC (cBioPortal).

cBioPortal was used to analyze the genetic alterations and correlations of SLC family genes in ccRCC. As shown in Figures 6, genetic alterations in SLC22A6, SLC22A7, SLC22A13, SLC25A4, SLC34A1, and SLC44A4 occurred in 42.2% of ccRCC patients, with SLC22A6 in 3%, SLC22A7 in 2.2%, SLC22A13 in 12%, SLC25A4 in 3%, SLC34A1 in 18%, and SLC44A4 in 4%. SLC22A6, SLC22A7, SLC25A4, and SLC44A4 were mainly altered by mRNA high, SLC22A13 was mainly altered by a deep deletion, and SLC34A1 was mainly altered by an amplification.

### 3.7. The Correlation between Different SLC Family Genes and Construction of PPI Network

**Figure 7:**
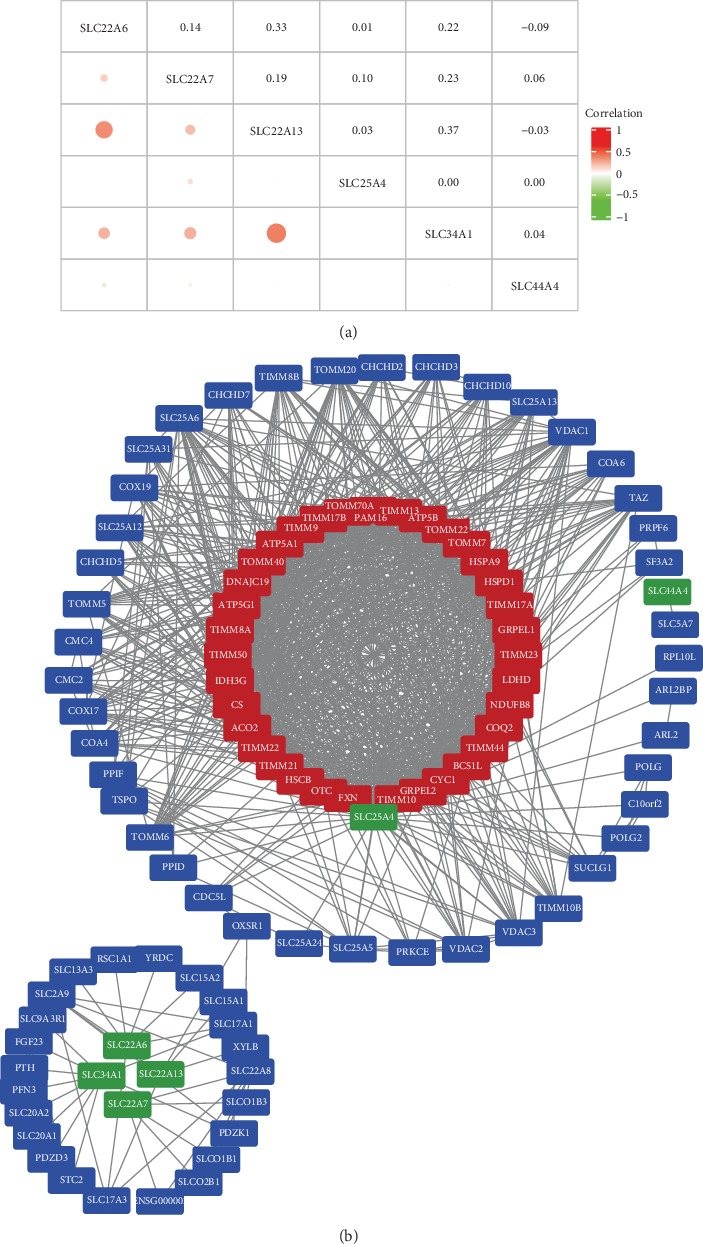
The correlation between different SLC family genes and the PPI network and module analysis. (a) The correlation between different SLC family genes in ccRCC. The size of the circle and the depth of the color represent the strength of the correlation. That is, the larger the circle, the darker the color, and the greater the correlation between genes. (b) The SLC family genes were used to construct the PPI network by STRING (https://string-db.org/), high confidence equals 0.700; the max number of interactors to show were 100). Total genes were mapped by Cytoscape. The MCODE was conducted to select appropriate modules of PPI network. Green indicated the six SLC family genes, red indicated the key modules, and blue indicated other interacting proteins.

We determined the correlations between SLC family genes by analyzing their mRNA expression levels (RNASeq V2 RSEM) via the cBioPortal online tool for ccRCC (TCGA, Provisional), and Pearson's correlation was included. The results indicated positive correlations between the following: SLC22A6 with SLC22A13 and SLC34A1; SLC22A7 with SLC22A13 and SLC34A1; and SLC22A13 with SLC34A1 (Figure 7(a)). In particular, there were significant positive correlations between SLC22A6 and SLC22A13 and SLC22A13 and SLC34A1.

Then, the SLC family genes were used to construct the PPI network by STRING. When building a PPI network, high confidence equals 0.700, and the max number of interactors to show was 100. After the PPI network was constructed, the network statistics are as follows: the number of nodes was 106, number of edges was 1053, average node degree was 19.9, avg. local clustering coefficient was 0.771, expected number of edges was 121, and PPI enrichment *P* value was 1.0*e*-16. Then, we use the Cytoscape tool to visualize the PPI network (Figure 7(b)). Finally, the Molecular Complex Detection (MCODE) plugins of Cytoscape were utilized to choose a hub cluster of the PPI network. The hub cluster contained 36 nodes and 561 edges. The genes concentrated in the hub cluster, such as translocase of inner mitochondrial membrane (TIMM) family genes, translocase of outer mitochondrial membrane (TOMM) family genes, and ATP family genes, were mainly involved in protein targeting to mitochondrion, mitochondrial transport, etc.

### 3.8. Functional Enrichment and KEGG Analysis of SLC Family Genes and Their Interacting Genes

**Figure 8:**
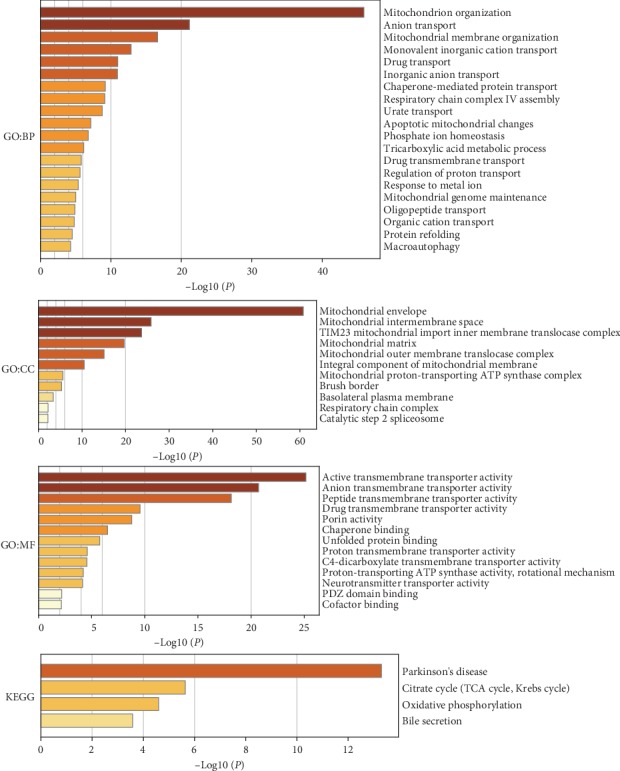
Functions of the SLC family genes and genes that interact with the SLC family genes were predicted with functional enrichment analysis and KEGG pathway analysis (Metascape). BP: biological processes; CC: cellular components; MF: molecular functions.

The functions of the SLC family genes and the genes significantly associated with SLC family gene alterations were predicted by GO and KEGG analyses with Metascape (Figure 8). The GO enrichment analysis predicted the functional roles of the target host genes on the basis of three aspects: biological process, cellular component, and molecular function. The biological process terms mainly included GO: 0007005 (mitochondrion organization), GO: 0006820 (anion transport), GO: 0007006 (mitochondrial membrane organization), GO: 0015672 (monovalent inorganic cation transport), and GO: 0015893 (drug transport); the cellular component terms mainly included GO: 0005740 (mitochondrial envelope), GO: 0005758 (mitochondrial intermembrane space), GO: 0005744 (TIM23 mitochondrial import inner membrane translocase complex), GO: 0005759 (mitochondrial matrix), and GO: 0005742 (mitochondrial outer membrane translocase complex); and the molecular function terms mainly included GO: 0022804 (active transmembrane transporter activity), GO: 0008509 (anion transmembrane transporter activity), GO: 1904680 (peptide transmembrane transporter activity), GO: 0015238 (drug transmembrane transporter activity), and GO: 0015288 (porin activity).

The KEGG can define the pathways related to the functions of the SLC family gene and their interacting genes (Figure 8). The relevant signaling pathways were hsa05012 (Parkinson's disease), M00009 (citrate cycle (TCA cycle, Krebs cycle)), hsa00190 (oxidative phosphorylation), and hsa04976 (bile secretion).

### 3.9. Validation of the Differential Expression of SLC Family Genes in ccRCC Using GSE53757 Datasets

**Figure 9:**
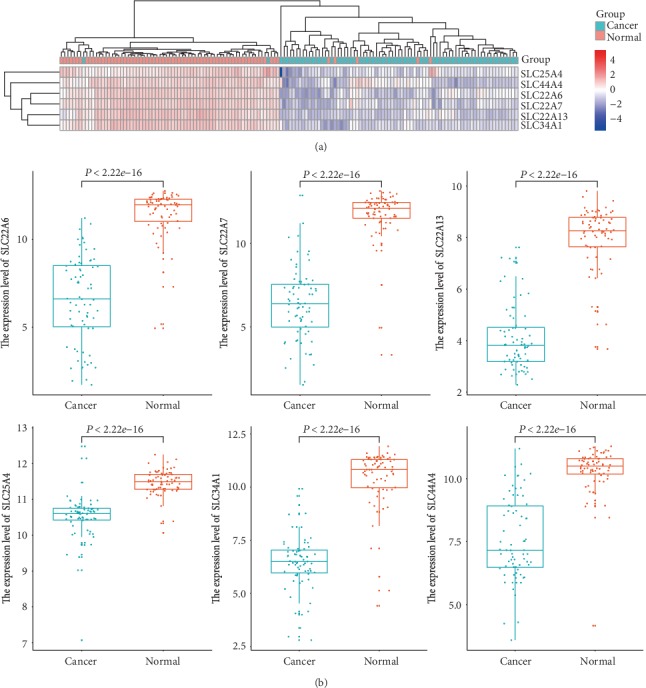
The GSE53753 dataset was used to reverify the differential expression level of the SLC family in ccRCC. (a) The heat map showed the expression of the SLC family in normal and cancer tissues. (b) The boxplot showed the expression of the SLC family in normal and cancer tissues. *P* < 0.01 meant the difference was statistically significant.

We downloaded the GSE53757 dataset from GEO. It contained 144 tissues, of which 72 are normal tissues and 72 are cancer tissues. Cancer tissue includes 24 cancer tissues in clinical stage 1, 19 cancer tissues in clinical stage 2, 14 cancer tissues in clinical stage 3, and 15 cancer tissues in clinical stage 4. After the difference analysis using the “limma” package, we found that the expression levels of SLC22A6, SLC22A7, SLC22A13, SLC25A4, SLC34A1, and SLC44A4 in ccRCC were significantly decreased (*P* < 0.01, as shown in Figures 9(a) and 9(b)), and the difference was statistically significant. Except for SLC25A4, the abundance difference of SLC22A6, SLC22A7, SLC22A13, SLC34A1, and SLC44A4 was more than 2-fold.

### 3.10. The Prognostic Value of the SLC Family Genes Was Reevaluated by the ICGC Dataset

**Figure 10:**
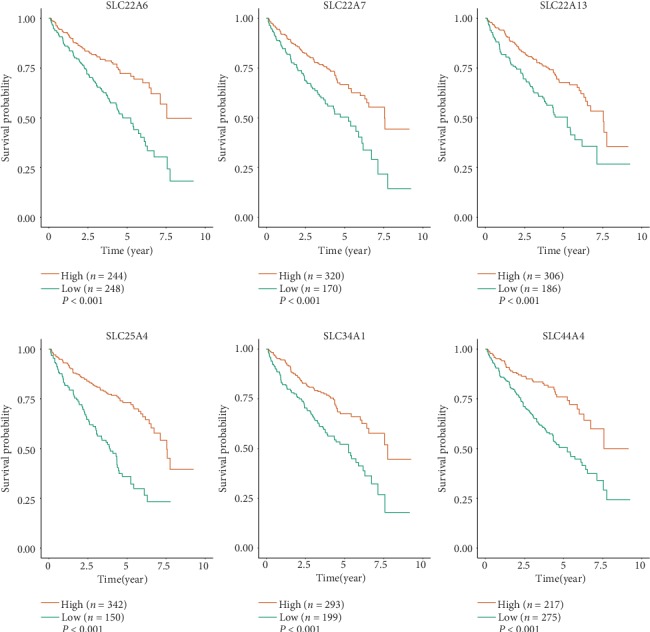
The overall survival of the ccRCC patients was reevaluated by the ICGC. *P* < 0.01 meant the difference was statistically significant.

We used the ICGC dataset to verify the prognostic value of the SLC family genes in ccRCC again. The cohort study included 492 patients, including 168 women and 324 men. As shown in Figure 10, the expression levels of SLC22A6, SLC22A7, SLC22A13, SLC25A4, SLC34A1, and SLC44A4 in ccRCC were significantly correlated with the OS, and the difference was statistically significant (*P* < 0.01). High SLC22A6, SLC22A7, SLC22A13, SLC25A4, SLC34A1, and SLC44A4 expression predicted improved OS. The analysis results were the same as before and confirmed that our previous analysis results were reliable.

## 4. Discussion

Metabolic disorders are hallmarks of cancer and offer the potential for cancer diagnosis, prognosis, and treatment [27–30]. Tumors readjust their metabolism to produce enough energy and substances for the proliferation of malignant cells. Recent studies have demonstrated that the pathological accumulation of metabolic intermediates such as fumarate and 2-hydroxyglutarate can contribute to tumorigenesis [31, 32]. Lipids and glycogen are abundant in ccRCC cells [33], suggesting that fatty acid and glucose metabolism changes during the development of ccRCC. Several studies have shown that metabolic changes play a key role in ccRCC progression [34], and other studies have revealed that poor patient survival is associated with the upregulation of pentose phosphate pathway and fatty acid synthesis pathway genes and the downregulation of the tricarboxylic acid (TCA) cycle genes [34, 35]. As a metabolic disease, many mutated genes, such as VHL, fumarate hydratase (FH), and succinate dehydrogenase (SDH), are involved in cellular respiration and energy metabolism [7]. HIF-1-induced genetic reprogramming promotes a classic Warburg phenotype in RCC through VHL or mechanisms dependent on metabolic enzymes [7, 36, 37]. Cancer cells predominantly produce energy by lactic acid fermentation, regardless of the oxygen level; this change was known as the Warburg effect or aerobic glycolysis [38, 39]. The loss of VHL leads to a HIF-1-dependent reprogramming of energy metabolism that includes elevated glucose uptake, glycolysis, and lactate production accompanied by a reciprocal decrease in respiration under aerobic conditions [36]. Multiple proteomics and metabolomics analyses have shown that compared with normal kidney tissue, the TCA cycle was downregulated between succinate and malate and upregulated between citrate and *α*-ketoglutarate in ccRCC tissues [40–44]. In addition, a combined proteomics and metabolomics study showed increased levels of metabolites in the glutamine and glutathione/oxidized glutathione pathways, including glutamine, glutamate, glutathione, and oxidized glutathione [41].

SLC22A6 was an organic anion/dicarboxylate exchanger. The outwardly directed gradient of *α*-ketoglutarate (*α*-KG) provides the driving force for the uptake of organic anions against the opposing force of the membrane potential [45]. Endogenous substrates of SLC22A6 include medium-chain fatty acids, *α*-ketoglutarate, citrulline [46], 5′-cyclic adenosine monophosphate (cAMP) and cyclic granulocyte-monocyte progenitor (cGMP) [47], prostaglandins E2 and F2, urate, and acid neurotransmitter metabolites [48]. SLC22A7 mediates cellular efflux of glutamate which may be transstimulated by uptake of some organic anions [49]. In addition to glutamate, SLC22A7 transports the following endogenous compounds into cells: glutarate [50], urate [51], purine and pyrimidine nucleobases, nucleosides and nucleotides, prostaglandin E2, prostaglandin F2, estrone-3-sulfate, allopurinol, and *α*-ketoglutarate [48, 52]. The endogenous compounds nicotinate, lactate, urate, succinate, and glutathione were identified as substrates of human SLC22A13. Transstimulation of urate uptake by succinate, lactate, and glutathione suggested that human SLC22A13 operates as an anion/anion exchanger [53].

Our study found that SLC22A6, SLC22A7, and SLC22A13 exhibited abnormal changes in various tumors. In ccRCC, the mRNA and protein expression levels of SLC22A6, SLC22A7, and SLC22A13 were significantly reduced compared to those in normal tissues. Further studies found that the expression levels of SLC22A6 and SLC22A13 were associated with the clinical stage, OS, and DFS. High SLC22A6, SLC22A7, and SLC22A13 expression predicts improved OS and DFS. Other genes in the SLC22 family, such as SLC22A1, SLC22A2, and SLC22A14, have not changed differentially in ccRCC. Low expression of SLC22A6 led to accumulation of *α*-KG in cells. *α*-KG was shown recently to activate 5′ adenosine monophosphate-activated protein kinase (AMPK) to promote anoikis resistance of tumor cells by enhancing its interaction with calcium/calmodulin-dependent protein kinase kinase 2 (CamKK2) [54]. In addition, glutamate dehydrogenase 1- (GDH1-) produced *α*-KG directly binds to and activates I-kappaB kinase beta (IKK*β*) and NF-*κ*B signaling, which promotes glucose uptake and tumor cell survival by upregulating glucose transporter 1 (GLUT-1) [55]. Low expression of SLC22A7 led to accumulation of glutamate. The upregulation of the glutamine and glutathione/oxidized glutathione pathways correlates with high grade, high stage, and metastasis of ccRCC [40, 41]. The released glutamine can act as a growth factor and a signal mediator in nonneuronal cancer tissues [56]. Low expression of SLC22A7 led to accumulation of lactate and glutathione. In ccRCC, the increase in GLUT-1 expression correlates with a decrease in the numbers of infiltrating CD8+ T cells [57], suggesting an additional mechanism by which ccRCC might suppress the immune system. This decrease in CD8+ T cells might be the result of increased lactate levels owing to GLUT-1 induction as lactate has been reported to inhibit T-cell activity [58].

In additional, SLC25A4, SLC34A1, and SLC44A4 exhibited abnormal changes in various tumors. In ccRCC, the mRNA expression levels of SLC25A4, SLC34A1, and SLC44A4 were significantly reduced. Further studies found that the expression levels of SLC25A4, SLC34A1, and SLC44A4 were associated with the clinical stage, OS, and DFS in ccRCC patients. High SLC25A4, SLC34A1, and SLC44A4 expression predicts improved OS and DFS. In ccRCC, the protein expression levels of SLC34A1 were significantly reduced, but the protein expression levels of SLC25A4 and SLC44A4 were not significantly different.

Inorganic phosphate (Pi) is associated with energy metabolism and acts as an integral part of signaling pathways by way of phosphorylation and dephosphorylation reactions of transcription factors and other intermediates of cellular signaling events [59, 60]. There was a consensus that tumor cells required relatively more phosphate because of their rapid rates of growth [61, 62]. In skin cancer, Pi was an essential nutrient for cell proliferation and for the promotion of tumorigenesis via the activation of the neuroblastoma RAS viral oncogene homolog (NRas) [63]. Transcellular transport of phosphate is initiated by several apically localized sodium-dependent Pi cotransporters (Na/Pi-cotransporters) that belong to the SLC20 (SLC20A1 and SLC20A2) and SLC34 (SLC34A1, SLC34A2, and SLC34A3) families [64]. All of the members are sodium-driven phosphate cotransporters that promote cell reabsorption of Pi [10]. In ccRCC, the mRNA and protein expression levels of SLC34A1 were significantly reduced. Theoretically, the decrease of SLC34A1 inhibited reabsorption of Pi, which led to the decrease of Pi in cells. In fact, the level of Pi in tumor cells was increased. In ccRCC, there was no differential expression of some transcellular transports responsible for Pi reabsorption in the GEPIA, such as SLC20A1 and SLC20A2, which rescued the reduced Pi reabsorption caused by SLC34A1. Therefore, we speculated that the functions of SLC34A1 are beyond Pi transmission. For example, although SLC20A1 was responsible for Pi transport, it was also responsible for activation by NF-*κ*B, an important protumoral pathway that might be induced by various cytokines [65].

In summary, we systematically analyzed the expression patterns and prognostic values of the SLC family in ccRCC and provided an improved understanding of the heterogeneity and complexity of the molecular biological properties of ccRCC. The results suggest that the downregulation of SLC22A6, SLC22A7, SLC22A13, SLC25A4, SLC34A1, and SLC44A4 in ccRCC may play an important role in the development of ccRCC. The low expression of SLC22A6, SLC22A13, SLC25A4, SLC34A1, and SLC44A4 can also be used as a molecular marker for identifying high-risk subgroups of ccRCC patients. The high expression of SLC22A6, SLC22A7, SLC22A13, SLC25A4, SLC34A1, and SLC44A4 can be used as a potential prognostic indicator for improving survival and prognosis in ccRCC patients.

## 5. Conclusions

Our study found that the expression levels of SLC22A6, SLC22A7, SLC22A13, SLC25A4, SLC34A1, and SLC44A4 were significantly lower in ccRCC tissues. They were significantly associated with clinical stage, OS, and DFS. They may be potential targets for the clinical diagnosis, prognosis, and treatment of ccRCC patients. However, findings should be validated with the clinical sample and functional experiment in the future.

## Figures and Tables

**Table 1:** Statistical table of tissues stained with SLC22A6, SLC22A7, SLC22A13, SLC25A4, SLC34A1, and SLC44A4. All slides were retrieved from the HPA. Compare the expression level in cancer and normal tissue from the aspects of staining, intensity, and quantity. P < 0.01 meant the difference was statistically significant.

		SLC22A6			SLC22A7			SLC22A13			SLC25A4			SLC34A1			SLC44A4		
		Normal	Cancer	*P* value	Normal	Cancer	*P* value	Normal	Cancer	*P* value	Normal	Cancer	*P* value	Normal	Cancer	*P* value	Normal	Cancer	*P* value
No. of patients		3	15		3	11		3	12		3	12		4	16		4	17	

No. of samples		6	49		3	21		6	47		3	23		6	43		6	38	

Gender	Male	2	8		2	6		1	6		2	6		3	8		3	8	
	Female	1	7		1	5		2	6		1	6		1	8		1	9	

Age (years)		41-73	56-89		16-70	52-77		1-41	52-77		41-73	56-80		1-73	59-89		1-61	52-80	

Staining	Not detected	0	13.5	*P* < 0.001	0	10	*P* = 0.005	0	11	*P* = 0.004	3	7	*P* = 0.295	0	12.5	*P* < 0.001	2	15.333	*P* = 0.237
	Low	0	1.5		0	1		0	0.5		0	5		0	2.5		0	0.667	
	Medium	1.5	0		3	0		0	0.5		0	0		4	0		2	1	
	High	1.5	0		0	0		3	0		0	0		0	0		0	0	

Intensity	Negative	0	13	*P* = 0.001	0	7	*P* = 0.011	0	11	*P* = 0.007	0	5	*P* = 0.295	0	10.5	*P* = 0.001	2	14.167	*P* = 0.275
	Weak	0	2		0	3		0	0		3	7		0	4		0	1.833	
	Moderate	1.5	0		3	1		0	0.5		0	0		2	0.5		2	1	
	Strong	1.5	0		0	0		3	0.5		0	0		2	0		0	0	

Quantity:	None	0	13	*P* = 0.005	0	7	*P* = 0.005	0	11	*P* = 0.001	0	5	*P* = 1.000	0	10.5	*P* = 0.016	2	14.167	*P* = 0.362
	<25%	0	0.5		0	4		0	1		3	2		2	2.5		0	1.167	
	25%-75%	1.5	0.5		3	0		1.5	0		0	2		0	2		2	0.667	
	>75%	1.5	1		0	0		1.5	0		0	3		2	0		0	1	

## Data Availability

The datasets used and analysed during the current study are available from the corresponding author on reasonable request.
