# Corrigendum to “The SLC Family Are Candidate Diagnostic and Prognostic Biomarkers in Clear Cell Renal Cell Carcinoma”

**DOI:** 10.1155/2020/1025178

**Published:** 2020-12-10

**Authors:** Weiting Kang, Meng Zhang, Qiang Wang, Da Gu, Zhilong Huang, Hanbo Wang, Yuzhu Xiang, Qinghua Xia, Zilian Cui, Xunbo Jin

**Affiliations:** ^1^Department of Urology, Shandong Provincial Hospital, Cheeloo College of Medicine, Shandong University, 324 Jingwuweiqi Road, Jinan, Shandong 250000, China; ^2^Department of General Surgery, The Second People's Hospital of Dongying, 28 Changchun Road, Dongying, Shandong 257000, China; ^3^Department of Human Resources, Shandong Provincial Hospital Affiliated to Shandong First Medical University, 324 Jingwuweiqi Road, Jinan, Shandong 250000, China; ^4^Department of Plastic Surgery, Jinan Central Hospital Affiliated to Shandong University, 105 Jiefang Road, Jinan, Shandong 250000, China; ^5^Department of Urology, Lanling People's Hospital, 4 Jiankang Street, Lanling, Shandong 277700, China; ^6^Department of Urology, Shandong Provincial Hospital Affiliated to Shandong First Medical University, 324 Jingwuweiqi Road, Jinan, Shandong 250000, China

In the article titled “The SLC Family Are Candidate Diagnostic and Prognostic Biomarkers in Clear Cell Renal Cell Carcinoma” [1], an affiliation was omitted in error. The affiliation “Department of Urology, Shandong Provincial Hospital Affiliated to Shandong First Medical University, 324 Jingwuweiqi Road, Jinan, Shandong 250000, China,” has been added to the affiliation list above as number (6) and it is affiliated to the authors “Hanbo Wang, Yuzhu Xiang, Qinghua Xia, Zilian Cui, and Xunbo Jin.”

The author “Weiting Kang” was affiliated to “Department of Urology, Shandong Province Hospital Affiliated to Shandong University, 324 Jingwuweiqi Road, Jinan, Shandong 250000, China,” which is incorrect. The correct affiliation for the author “Weiting Kang” is “Department of Urology, Shandong Provincial Hospital, Cheeloo College of Medicine, Shandong University, 324 Jingwuweiqi Road, Jinan, Shandong 250000, China.”

The author “QiangWang” was affiliated to the “Department of Human Resources, Shandong Province Hospital Affiliated to Shandong University, 324 Jingwuweiqi Road, Jinan, Shandong 250000, China,” which is incorrect. The correct affiliation for the author “QiangWang” is “Department of Human Resources, Shandong Provincial Hospital Affiliated to Shandong First Medical University, 324 Jingwuweiqi Road, Jinan, Shandong 250000, China.”

The corrected list of affiliations is shown in the author information above.
